# Nasogastric tube insertion in anesthetized and intubated patients: a new and reliable method

**DOI:** 10.1186/1471-230X-12-99

**Published:** 2012-08-01

**Authors:** Yung-Fong Tsai, Chiao-Fen Luo, Amina Illias, Chih-Chung Lin, Huang-Ping Yu

**Affiliations:** 1 Department of Anesthesiology, Chang Gung Memorial Hospital, 5 Fu-Shin Street, Kwei-Shan, Tao-Yuan, Taiwan, 333, R.O.C; 2 College of Medicine, Chang Gung University, 259 Wen-Hwa 1st Road, Kwei-Shan, Tao-Yuan, Taiwan, 333, R.O.C; 3 Graduate Institute of Clinical Medical Sciences, Chang Gung University, 259 Wen-Hwa 1st Road, Kwei-Shan, Tao-Yuan, Taiwan, 333, R.O.C

**Keywords:** Nasogastric tube insertion, Intubation stylet, Highwayman’s hitch, Success rate

## Abstract

**Background:**

The “Rusch” intubation stylet is used to make endotracheal tube intubation easy. We designed this study to evaluate the usage of this equipment in the guidance of nasogastric tube (NGT) insertion.

**Methods:**

A total of 103 patients, aged 23 to 70 years, undergoing gastrointestinal or hepatic surgeries that required intraoperative NGT insertions were enrolled into our study. The patients were randomly allocated to the control group (Group C) or the stylet group (Group S) according to a computerized, random allocation software program. In the control group, the NGT was inserted with the patient’s head in an intubating position. In the stylet group, the NGT was inserted with the assistance of a “Rusch” intubation stylet tied together at the tips by a slipknot. The success rates of the two methods, the durations of the insertions, and the occurrences of complications were recorded. All of the failed cases in the control group were subjected to the new technique used in the stylet group, and the successful rescue rate was also evaluated.

**Results:**

Successful insertions were recorded for 52/53 patients (98.1%) in Group S and for 32/50 patients (64%) in Group C. The mean insertion times were 39.5 ± 19.5 seconds in Group C and 40.3 ± 23.2 seconds in Group S. Successful rescues of failure cases in Group C were achieved in 17/18 patients (94.4%) with the assistance of a “Rusch” intubation stylet.

**Conclusions:**

The “Rusch” intubation stylet-guided method is reliable with a high success rate of NGT insertion in anesthetized and intubated patients.

**Trial registration:**

Institutional Review Board of Chang Gung Memorial Hospital (IRB: 98-2669B) and Australian New Zealand Clinical Trials Registry (ACTRN12611000423910)

## Background

Inserting a nasogastric tube (NGT) into a paralyzed and intubated patient is sometimes difficult and frustrating. A potentially difficult insertion is hard to predict according to the outward appearance of the patients before NGT placement. An average failure rate of nearly 50-66% was reported on first attempt made by conventional method with the patient’s head in an intubating position [1,2]. After several unsuccessful insertions, the incidences of nasal mucosal bleeding [3,4] and unstable vital signs (hypertension, tachycardia, and arrhythmias) usually increases [5].

It has been acknowledged that most difficulties in NGT insertions are due to anatomic reasons [6]. The most common sites of impaction are the piriform sinus, the arytenoids cartilage [6,7], and the esophagus, which becomes compressed by the inflated cuff of an endotracheal tube. Another important issue concerns the material properties of the NGT. The NGT is usually made of polyurethane, which makes the NGT soft and less traumatic [8]. It is not easy to guide the tube when using a small, soft, or long caliber instrument and the NGT tends to coil or kink when encountering an anatomic block. After a failed passage, the NGT is warmed by body heat and becomes softer and more likely to coil during the next attempt. A silicone stomach tube is more pliable and thus more difficult to insert [8]. Additionally, there are four non-opposing lateral eyes on the distal part of a common NGT. These eyes result in an incomplete caliber, as there are weak points on the distal end. The end of the NGT is easy to bend, and can kink when passing through an indirect or narrow passage or tunnel. Sometimes the NGT is already slightly folded by the package or is compressed by the outer caliber segments rolled up in a storage bag which also contributes to weak points during placement.

We introduce a new and simple technique with a high success rate for NGT insertions overall and for first attempts in particular. We inserted a NGT with the assistance of a “Rusch” intubation stylet tied together at the tip by a slipknot- Highwayman’s hitch (also called a Draw hitch). The main feature of this slipknot is that it can be untied with a very light tug of the distal end, allowing for a quick release. The skills necessary are simple, and the method directs the NGT from the chosen nostril to nearly the inlet of the stomach, bypassing the obstacles between these two points.

This study was designed to investigate whether the “Rusch” intubation stylet-guided method would facilitate the passage of the NGT in anesthetized and intubated patients.

## Methods

### Trial design

This prospective, randomized, and double-blinded study was conducted in patients scheduled for gastrointestinal or hepatic surgeries, and required an intraoperative NGT insertion. It was approved by the Institutional Review Board of Chang Gung Memorial Hospital (IRB: 98-2669B), and the Australian New Zealand Clinical Trials Registry (ACTRN12611000423910), and was conducted according to the Declaration of Helsinki. Written informed consent was obtained from each patient before entering the operation room.

### Participants

Patients who were younger than twenty years old or older than seventy years old or who had coagulopathy (abnormal prothrombin time, partial thromboplastin time, and platelet disorders), nasal stenosis, nares obstruction, nasal septal deviation, upper respiratory tract diseases or anomalies, or esophageal disorders were all excluded from this study. Patients who met the criteria for difficult intubation (Cormack and Lehane and/or Mallampati scores of 3 or 4) were also excluded.

### Interventions

All of the patients underwent general anesthesia and endotracheal tube intubation before a NGT insertion. General anesthesia was induced with fentanyl at 2 μg/kg, lidocaine at 0.5 mg/kg, propofol at 2 mg/kg, and cisatracurium at 0.2 mg/kg intravenously. All patients were tracheally intubated, with a 7.0 mm internal diameter endotracheal tube in females and a 7.5 mm internal diameter endotracheal tube in males. Anesthesia was maintained by sevoflurane at an end-tidal concentration of one minimum alveolar anesthetic concentration.

A single attending anesthesiologist was responsible for all NGT placements. Our attendant had practiced the two methods of NGT insertions for two weeks (20 patients in each group) before our study began. After the end-tidal concentration of sevoflurane was found to be more than one minimum alveolar anesthetic concentration and after all twitches responses to a train-of-four nerve stimulator had disappeared, our attendant would gently prepare the patients’ nostrils with a cotton stick dipped in 2% lidocaine jelly. The nostril that was more patent and did not have nasal obstruction or septal deviation was chosen for NGT insertion. The endotracheal tube was fixed with tape at the corner of the mouth opposite the chosen nostril.

**Figure 1:**
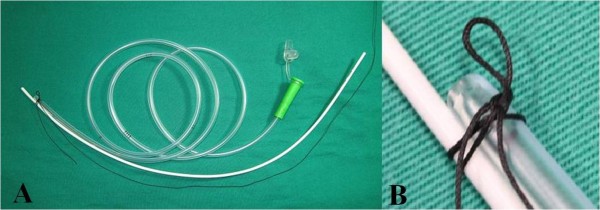
**(A) “Rusch” intubation stylet is tied to nasogastric tube by a Highwayman**’**s hitch.** (**B)** A magnified picture of the slipknot (Highwayman’s hitch). A Fr.14 nasogastric tube and a 2.6 mm “Rusch” intubation stylet were tied together at the tip by a slipknot (Highwayman’s hitch) with a surgical silk suture (70 cm in length, size 3–0).

Water-soluble lubricant jelly (3 mL) was poured into the chosen nostril. For Group C patients, a Fr.14, 125-cm NGT was inserted with the patient’s head in an intubating position. For Group S patients, a same-sized NGT was introduced with the assistance of a 2.6 mm “Rusch” intubation stylet tied together at the tip by a slipknot (named Highwayman’s hitch or Draw hitch) with a surgical silk suture (70 cm in length, size 3–0) (Figure 1). The patient’s head was also kept in an intubating position during NGT insertion in Group S. The tip of the “Rusch” intubation stylet was tied extending from the tip of the NGT (about 1.0 cm protruding) to reduce resistance during nasal threading. Stylet navigation through the nasal cavity and directly into the esophagus is made smoother and easier by guiding the stylet in a lateral direction after it passes through the nasal cavity. When the “Rusch” intubation stylet was introduced to its deepest depth, the slipknot was untied and withdrew with the “Rusch” intubation stylet smoothly.

We determined the necessary NGT length required to reach the stomach by measuring the distance from the patient’s xiphoid process to the closer earlobe via the nose [9]. The start time was defined as when the NGT was inserted into the nostril. The end time was defined as the time after the successful completion of the NGT insertion procedure or the time after two failed attempts. The duration of insertion time was measured with a stopwatch. A successful insertion was defined as hearing the gurgling sounds of auscultation over the epigastrium when 20 mL of air was injected via the NGT, or as observing the aspiration of gastric content from the NGT.

If the first attempt failed, another attempt involved a second insertion with the original but cleaned NGT by the same procedure. It was considered a failed insertion if both of these attempts were unsuccessful. The failed cases in Group C were rescued by the technique used in Group S. In Group S, the failed intubations were rescued through the assistance of a laryngoscope with a Magil forcep.

### Outcomes

We analyzed the overall success rate (succeeded within two attempts), success rate on first attempt, failure rate, and the duration of insertion time in both groups. We also assessed the successful rescues of failure cases in Group C by using this novel method. The complications of NGT passage, such as bleeding, kinking, knotting, or tracheal insertion were recorded.

### Randomization and blinding

All of the patients enrolled into this study were randomly allocated into two groups (the control group and the stylet group) according to a computerized, random-allocation software program [10]. The patient did not know which group he or she was enrolled in. After the patient was anesthetized, the performer of the NGT placement was informed of which group the patient was enrolled in according to a computerized, random-allocation software program. The authors did not perform any NGT insertion to avoid operator bias. An observer was responsible to judge whether the attempt was a success or failure, and whether any complication occurred. The observer was also blind to the groups.

### 2.6-mm “Rusch” intubation stylet, Germany

The “Rusch” intubation stylet used in this study was 2.6 mm in diameter and 40.5 cm in length, and it is normally used as an introducer for tracheal intubation. It is a readily available stylet equipped in our anesthesiology department and intensive care units. The tip of the stylet is malleable and flexible, with a rubber outer sleeving. The tip is easy to bend around the obstructions and causes less mucosal trauma. When the stylet is tied to the NGT, it is easy to introduce the NGT through the nasal cavity, oropharyngeal space, and upper esophageal sphincter to nearly above the gastroesophageal junction.

### Highwayman’s hitch

The highwayman’s hitch is one type of slipknot (Figure 1). It is an insecure, quick-release knot. The main feature of the knot is that it can be untied with a very light tug of the distal working end, allowing for a quick release [11]. Here we introduce the instructions for how to tie a highwayman’s hitch step-by-step.

### Instructions for tying a Highwayman’s hitch

 1. Begin with a single loop (bight) in a silk suture behind both the NGT and stylet tip ends.

 2. The left side of the loop will be the standing end.

 3. The right side of the line will be the release end in order to be untied, and the release end must be as long as the stylet.

 4. Take the standing end (left end) and form another loop.

 5. Tuck this loop into the original loop.

 6. Take the release end (right end) of the silk suture and form another loop.

 7. This loop will go inside the last loop made.

 8. At this step, tighten the knot by pulling on the standing end.

 9. Pulling on the standing end will lock in the quick release portion of the knot.

 10. Tug on the release distal end for a quick release.

### Sample size

The sample size was calculated using Gpower 3.1.2 software. A pilot study of 15 cases per group suggested an approximate 27% improvement (from base rate of 60% to 87%) in success rate using these techniques. Consequently, a power calculation (α = 0.05 and β = 0.2) indicated a minimum of 41 patients for each group.

### Statistical methods

Data were analyzed with SPSS v.15 (SPSS Inc., Chicago, IL, USA). Demographic data were analyzed by the two-tailed t-test and Pearson Chi-Square test. The time of NGT insertion was analyzed by the two-tailed t-test, and the complication rates of NGT insertion were analyzed by the Pearson Chi-Square test and Fisher’s exact test. A value of p <0.05 was considered to be statistically significant.

## Results

### Study population

One hundred and three patients were enrolled into this study. There were no statistically significant differences in the demographic data (age, gender, height, weight, and ASA physical status classification) between the two groups (Table 1).

**Table 1:** Patient characteristics in each group

	**Control group (n = 50)**	**Stylet group (n = 53)**	**p**
Age (yr)	54.3 ± 10.076	55.38 ± 10.457	0.596
Height (cm)	162.224 ± 8.428	160.900 ± 9.293	0.451
Weight (kgw)	63.318 ± 11.605	62.342 ± 12.990	0.689
Gender (male/female)	30/20	28/25	0.463
ASA class, n (%)	2.22 ± 0.507	2.28 ± 0.495	0.525
I	2 (4)	1 (1.9)	
II	35 (70)	36 (67.9)	
III	13 (26)	16 (30.2)	

Values are presented as mean and SD or number.

ASA class = American Society of Anesthesiologists’ Physical Status Classification.

### Participant flow

**Figure 2:**
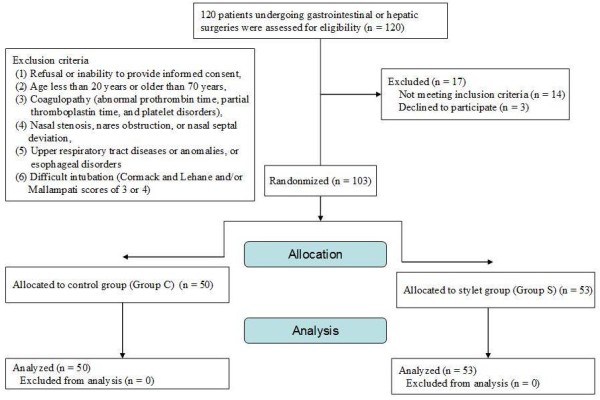
The flow of the participants in the study.

The flow of the participants in the study is summarized in Figure 2.

### Outcomes

**Figure 3:**
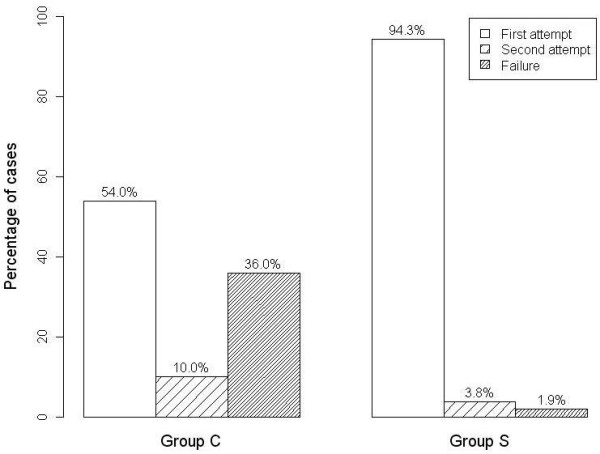
**The success rates of nasogastric tube insertion.** Group C = control group; Group S = stylet group. Data are shown as percentages of cases in each group. The p values were <0.001 for both the first attempt and the overall success rate vs. the control group.

The success rate of NGT insertion was 32/50 patients (64%) in Group C and 52/53 patients (98.1%) in Group S (Figure 3), and there was a significantly higher success rate in Group S than in Group C (p < 0.01). In Group C, 27/50 (54%) and 5/50 (10%) patients had successful NGT insertions on the first and second attempts, respectively. In Group S, 50/53 (94.3%) and 2/53 (3.8%) patients had successful NGT insertions on the first and second attempts, respectively. There were three failed NGT placements on the first attempt in Group S. One insertion failed because the highwayman’s hitch unexpectedly untied during the procedure. Reinsertion of the NGT was achieved on the second attempt. The remaining failed case was then rescued with the help of Magil forceps during a laryngoscope.

Successful rescues of failure cases in Group C were achieved in 17/18 patients (94.4%) by using this new technique with the assistance of an intubation stylet. Of these, 16/18 (88.9%) and 1/18 (5.56%) patients had successful NGT insertions on the first and second rescue attempts, respectively.

The mean insertion time was 39.5 ± 19.5 s in the control group and 40.3 ± 23.2 s in the stylet group (Table 2). There was no statistical difference in insertion time between the two groups (p = 0.373).

**Table 2:** Total time for insertion and complications

	**Control group (n = 50)**	**Stylet group (n = 53)**	**p**
**Total time for insertion (s)**	39.5 ± 19.5	40.3 ± 23.2	0.854
**Complication, n (%)**			
**Kinking**	9 (18)	0 (0)*	0.001
**Bleeding**	6 (12)	6 (11.3)	0.914

* Significant versus control group.

Kinking of the NGT and nasal mucosal bleeding were the most common complications. Kinking of the NGT occurred in 9 patients (18%) in Group C but in no patients in Group S, resulting in a significantly higher kinking rate in Group C compared to Group S (p = 0.0001). Nasal mucosal bleeding occurred in 6 patients (12%) in Group C and also in 6 patients (11.32%) in Group S. There was no statistical difference between the two groups (p = 0.914).

### Adverse events

Throughout the study, the NGT insertions guided with a 2.6-mm “Rusch” intubation stylet were generally safe. No major adverse event developed in any patients.

## Discussion

Inserting a nasogastric tube into anesthetized and intubated patients is sometimes very difficult and traumatic. After several failed attempts, complication rates usually increase. Threading the pliable NGT through probable anatomic obstacles without any manipulations or facilities is challenging.

Therefore, some authors suggest the compression of the ipsilateral lateral neck at the level and lateral border of the thyrohyoid membrane to transiently collapse the ipsilateral piriform sinus and slightly move the arytenoids cartilage so that the NGT can more easily pass through via the lateral or posterior hypopharynx [6]. Deflation of the cuff of the endotracheal tube can release the compression over the esophagus and improve NGT passage. The methods adopted, which have high success rates, include the use of a slit endotracheal tube placed via the nasoesophageal route [2], a laryngoscope with a Magil forcep [12], a GlideScope for placement [13], or gloved finger steering to navigate the NGT [14]. However, these methods may be difficult in patients with limited mouth opening and cervical spine injuries, and some of these methods may be time-consuming in preparation or performance. Other authors suggest forward neck flexion [15], head rotation [1], or forward displacement of the larynx [7] to facilitate the threading of the NGT more smoothly through lateral or posterior hypopharynx spaces; the NGT can then enter the esophageal opening [1,6,14]. Gupta D et al. [16] suggests inflation with air via a facepiece to open the upper esophageal sphincter.

Considering the faults of NGT’s material properties, some authors suggest stiffening the NGT before an insertion [3,17-20]. The suggested methods include immersion of the NGT in ice-cold water [17], keeping the NGT in a refrigerator [18], using a water-fill method [3], freezing the NGT with distilled water [4], choosing a large-caliber NGT [8], or introducing guidewire [21], forcep [19], or guitar string [20] into the NGT before use. These steps significantly reduce NGT kinking and improve the success rate of insertion.

The use of a slit endotracheal tube may cause obvious mucosal damage and bleeding [2]. Deflation of the cuff of the endotracheal tube, freezing the NGT with distilled water [4], and the water-fill method are of concern in patients who have not fasted to avoid pulmonary aspiration or regurgitation. The insufflation of air in the oropharynx might possibly lead to regurgitation and aspiration despite the presence of a cuffed tracheal tube and adequate starvation [16]. Forward displacement of the larynx occasionally causes bradycardia via vasovagal reflex due to compression of the bilateral carotid arteries [22]. Forward neck flexion sometimes causes increased peak pressure of air way when the endotracheal tube bends. Our novel method is free from these limitations.

In our study, the success rate of NGT intubation in Group S on first attempt was significantly higher than that in Group C (94.3% vs. 54%, p < 0.01). The highwayman’s hitch is used to bind together the tips of the NGT and the “Rusch” intubation stylet on their distal ends (introduced to the lower esophagus), and it can be quickly released with a very light tug of the proximal end (outside of the nostril). Tying a highwayman’s hitch is easy to learn, and both the “Rusch” intubation stylet and the surgical silk sutures (70 cm in length, size 3–0) are readily available in operation rooms.

In the control group, successful rescues of failed cases were achieved in 17/18 patients (94.4%) by using this new technique with an intubation stylet as an introducer. Sixteen patients were rescued successfully on the first attempt. We recommend this novel technique not only due to the high success rate on the first attempt in common cases, but also because of the high rescue rate for difficult cases. Because of the limited number of rescue cases in our study, further studies will need to evaluate this outcome.

The mean insertion time was 39.5 ± 19.5 s in Group C and 40.3 ± 23.2 s in Group S. There was no statistical difference in the mean insertion time between the two groups. The insertion time was defined as the procedure of intubation and did not include the time needed to tie the highwayman’s hitch. If this was included, the insertion time in Group S would be a little longer. However, it can take only a few seconds to tie a highwayman’s hitch with practice.

A total of 18 of the 103 study patients developed complications. Kinking of the NGT occurred in 9 patients (18%) in Group C but in no patients in Group S. Using a 2.6-mm “Rusch” intubation stylet as an introducer makes it easy to guide the NGT through sites of impaction and up to 40 cm deep into the nostril without any kinking or knotting. Insertions using smaller size NGTs or softer silicone stomach tubes have kinking more often, and these may particularly benefit from the usage of this method.

Another common complication was nasal mucosal bleeding. This occurred in 6 patients (12%) in Group C and also in 6 patients (11.3%) in Group S. All of these complications involved mucosal blood tinged, not active bleeding, and no blood entered the mouth. None of these patients needed further medical or surgical treatment. Although the NGT tied with a 2.6-mm “Rusch” intubation stylet in Group S had a larger diameter, it did not cause more complications of nasal mucosal bleeding than the single NGT used in Group C. In some studies, the patients’ nostrils were prepared with vasoconstrictors to lessen the occurrences of bleeding. We did not use any vasoconstrictors to prepare the nostrils of patients in this study. Several reports have revealed other complications (knotting and tracheal insertion) during NGT insertion [23]. The incidences of knotting and tracheal insertion were not observed in our study, and this might be due to inadequate sample size to evaluate these complications.

If the “Rusch” intubation stylet is replaced by a fiberoptic scope for guiding NGT insertion, physicians would benefit from direct vision of the procedure. However, comparisons with a firberoptic-guided method using a slipknot to insert a NGT still require further investigation.

The average patient height in our study was 160 cm (range from 140 to 179 cm). The “Rusch” intubation stylet used in this study was only 40.5 cm in length. It is possible that in taller patients, the length of the stylet might not be sufficient to reach the gastroesophageal junction. The potential success rate of the technique on taller patients is unknown.

Some reliable methods need to open patient’s mouth including a slit endotracheal tube, a laryngoscope with a Magil forcep, a GlideScope for placement, and gloved finger to navigate the NGT [2,13,15,16]. Our method does not require oral manipulations. In this regard, our technique might have potential role on patients with limited mouth opening or other difficult airways. Additional studies are needed to precisely elucidate whether our method had beneficial effects on those patients.

## Conclusions

The “Rusch” intubation stylet-guided method has a high success rate of NGT insertion in anesthetized and intubated patients. There was no kinking of the NGT with this method, and the incidences of bleeding and the mean time of insertion were not statistically greater than those in the control group. It proved to be efficient in facilitating NGT insertion in difficult cases. We recommend the use of this novel technique not only for routine performances, but also for the rescues of difficult placements.

## Abbreviations

NGT, Nasogastric tube; Group C, Control group; Group S, Stylet group.

## Competing interests

The authors declare that they have no competing interests.

## Authors’ contributions

LCF, IA, LCC, and TYF participated in the design of the study. YHP and TYF conceived of the study, and participated in its design and coordination and helped to draft the manuscript. All authors read and approved the final manuscript.

## Pre-publication history

The pre-publication history for this paper can be accessed here:

http://www.biomedcentral.com/1471-230X/12/99/prepub
